# OTUD1 stabilizes PTEN to inhibit the PI3K/AKT and TNF-alpha/NF-kappaB signaling pathways and sensitize ccRCC to TKIs

**DOI:** 10.7150/ijbs.68980

**Published:** 2022-01-24

**Authors:** Wentao Liu, Bin Yan, Haixin Yu, Jiannan Ren, Mou Peng, Liang Zhu, Yinhuai Wang, Xin Jin, Lu Yi

**Affiliations:** 1 Department of Urology, The Second Xiangya Hospital, Central South University, Changsha, Hunan, 410011, China; 2 2Cancer center, Union Hospital, Tongji Medical College, Huazhong University of Science and Technology, Wuhan, 430022, China; 3 3Uro-Oncology Institute of Central South University, Changsha, Hunan, 410011, China; 4 4Hunan Engineering Research Center of Smart and Precise Medicine, Changsha, Hunan, 410011, China; 5 5These authors contributed equally.

**Keywords:** ccRCC, OTUD1, PTEN, TKIs

## Abstract

Clear cell renal cell carcinoma (ccRCC) is the most common subtype of renal cell carcinoma and has the highest mortality rate. For metastatic RCC, systemic drug therapy is the most important method in addition to surgical tumor reduction. In recent years, tyrosine kinase inhibitors (TKIs) targeting the angiogenesis have been applied to treat ccRCC and achieved profound therapeutic effects. It has been reported that most patients receiving antiangiogenic therapy will develop resistance within 15 months. The mechanism of resistance to targeted therapy is extremely complex and has not been clarified. Ovarian tumor-associated protease domain-containing proteins (OTUDs) belonging to DUBs play a critical role in the tumorigenesis of solid tumors. However, the specific role of OTUDs in ccRCC is still elusive. Here, we investigated the clinicopathological role of OTUD family members in ccRCC. We demonstrated that OTUD1 was downregulated in renal cancer and involved in the poor prognosis of renal cancer. Then, we showed that OTUD1 inhibits cancer cell growth. Moreover, analysis of OTUD1 RNA-seq data indicated that OTUD1 inhibition triggers the AKT and NF-kappa B pathways in renal cancer cells. Furthermore, OTUD1 interacts with PTEN and regulates its stability. Subsequently, we revealed that downregulation of OTUD1 contributes to the sensitivity of renal cancer cells to TKIs, and this effect was blocked by TNF/NF-kappa B inhibitors and AKT inhibitors. Thus, we identified that the OTUD1-PTEN axis suppresses tumor growth and regulates the resistance of renal cancer to TKIs.

## Introduction

Renal cell carcinoma (RCC) is a common urologic malignancy 1. Its incidence and mortality rates rank fifteenth among those of all malignant tumors 2. Clear cell renal cell carcinoma (ccRCC) is the most common subtype of RCC and has the highest mortality rate 3. According to clinical stage, RCC is divided into localized RCC (T1-T2), locally advanced RCC (T3-T4) and metastatic RCC (M1); approximately 16% of RCCs are metastatic 2. The 5-year survival rate of localized RCC is approximately 93%, while that of metastatic RCC is only 12% 2. Surgical resection is the main treatment for RCC 4. For metastatic RCC, systemic drug therapy is the most important method in addition to surgical tumor reduction 5. In recent years, targeted drugs and immune checkpoint inhibitors have been applied to treat ccRCC and have achieved profound therapeutic effects 6. Exploration of the mechanism by which ccRCC develops is crucial for identifying new targets to prolong the survival time of patients with ccRCC.

The von Hippel-Lindau (VHL) gene mutation (present in approximately 90% of renal cancer patients) results in the excessive vascularization of tumor tissues 7. Antiangiogenesis therapy, including VEGF (bevacizumab), tyrosine kinase inhibitors (TKIs), and mTOR inhibitors (everolimus), has become the standard therapy for patients with renal cancer 8. It has been reported that most patients receiving antiangiogenic therapy will develop resistance within 15 months 9. The mechanism of resistance to targeted therapy is extremely complex and has not been clarified.

Ubiquitination is a reversible process regulated by the ubiquitin-proteasome system, which is important for the homeostasis of cells 10. Deubiquitinating enzymes (DUBs) remove the ubiquitination modification from the target protein and play an important role in the ubiquitin-proteasome system. DUBs are closely correlated with the sensitivity of antitumor drugs 11. Ovarian tumor-associated protease domain-containing proteins (OTUDs) belonging to DUBs play a critical role in the tumorigenesis of solid tumors 12. However, the specific role of OTUDs in ccRCC is still elusive. In the current study, we investigated the clinicopathological role of OTUD family members in ccRCC. We showed that OTUD1 was closely associated with an unfavorable prognosis in ccRCC. Then, we found that OTUD1 participates in inhibiting cell proliferation and inactivating the PI3K/AKT and TNF-alpha/NF-kappa B signaling pathways in ccRCC. Moreover, we revealed that OTUD1 stabilizes PTEN, which is a negative regulator of both the PI3K/AKT and TNF-alpha/NF-kappa B signaling pathways 13. We proved that OTUD1 regulates the resistance of TKIs in ccRCC via the NF-kappa B pathway. Therefore, we showed that OTUD1 loss contributes to the resistance of TKIs through PTEN in ccRCC.

## Methods and material

### Cell lines and cell culture

The two ccRCC cell lines that we cultured, 786-O and ACHN, were obtained from Yuchi Biology (Shanghai, China), and both cell lines were identified by short tandem repeat (STR) profiling. Cells were cultured in RPMI-1640 medium (Gibco, USA) or minimum essential medium (MEM, Gibco, USA) supplemented with 10% fetal bovine serum (FBS; AC03L055, Shanghai Life-iLab Biotech, China) and 1% penicillin, placed in an incubator at 37 °C in 5% CO2.

### Antibodies, chemical agents, siRNAs and plasmids

The antibodies used for western blot analysis are as follows: GAPDH (#60004-1-Ig, Proteintech, 1:5000 dilution), OTUD1 (#bs-17563R, Bioss antibodies, 1:2000 dilution), PTEN (#22034-1-AP, Proteintech, 1:1000 dilution), AKT (#bsm-33278M, Bioss antibodies, 1:2000 dilution), pAKT-S473 (#4060S, Cell signaling technology,1:1000 dilution), cleaved caspase 3 (#9661, Cell signaling technology,1:2000 dilution), Caspase 3 (#19677-1-AP, Proteintech, 1:1000 dilution). MG132 (#S2619), Sunitinib (#S7781), Pazopanib (#S3021), JSH-23 (#S7351), MK2206 (#S1078), GSK1120212 (#S2673), and LY294002 (#S1105) were purchased from Selleckchem (Shanghai, China). HA-OTUD1 was constructed by cloning the cDNA of OTUD1 into the OmicLinkTM Expression Clone (CMV Promoter) (GeneCopoeia, EX-V0006-M14, USA). The siRNAs were purchased from RiboBio. The sequences of the siRNAs were provided in Table S1. Cells were transfected with the indicated plasmids or siRNAs using Lipofectamine 2000 (Thermo Fisher Scientific, China) according to the manufacturer's instructions. Caspase 3 activity assay kit (#ab39401) was purchases from Abcam.

### Coimmunoprecipitation and Western blot analysis

The detail of coimmunoprecipitation (IP) was reported previously 14. In brief, cell proteins were lysed in the RIPA buffer (#P0013, Beyotime, China) and added with protein A+G beads (#P2029, Beyotime, China) and IgG (#A7007, Beyotime, China) or a primary antibody. The next day, the beads were washed 6 times with RIPA buffer, and 60 µl of 1X loading buffer was added. Finally, the beads were boiled and subjected to Western blotting. For Western blotting analysis, the boiled protein lysates were electrophoresed on SDS-PAGE gels. Protein expression levels were measured by using ImageJ software (National Institutes of Health, USA).

### Real time RT-PCR analysis

Total RNA from cells was extracted using TRIzol reagent (#AG21102, Accurate Biotechnology, Hunan, China). The reverse transcription kits (#AG11728, Accurate Biotechnology, Hunan, China) and PCR kits (#AG11701, Accurate Biotechnology, Hunan, China) was used to perform RT-qPCR according to the manufacturer's instructions. All values were normalized to the corresponding GAPDH values, and the 2^-ΔΔ^Ct method was used to quantify the fold change. The sequences of primers were provided in Table S2.

### Cell proliferation assay

For Cell Counting Kit-8 (CCK-8) assay, CCK-8 reagent (#C0037, Beyotime) was added to each cell well and an absorbance of 450 nm was measured with a microplate reader.

All animal experiments were approved by the ethics committee of the Second Xiangya Hospital, Central South University (Animal license number: 2021897). BALB/c nude mice (6 weeks old) were purchased from Vital River (Beijing, China). Cells were subcutaneously injected into the left side of the backs of the mice (1 × 10^7^ cells per mouse). Tumor volume was calculated using the formula (L × W^2^)/2. Once the mice were euthanized, the tumors were excised and weighed.

### Tissue microarray and immunohistochemistry (IHC)

The tissue microarray slides (# U081ki01) were purchased from bioaitech, China. The tissue microarray specimens were immunostained with OTUD1 and PTEN. The method of scoring of staining intensity was mentioned previously 14, 15.

### Cell cycle and Annexin v-FITC/PI assay

Cells were washed three times with PBS and resuspended in Binding Buffer. For cell cycle analysis, cells were stained with Propidium iodide (PI). For Annexin v-FITC/PI assay, cells were stained with Annexin v-FITC and PI following the manufacturer's instruction of Annexin V-FITC Apoptosis Detection Kit (Solarbio life science, Beijing, China). Cells were incubated for 15 min at room temperature and analyzed on a flow cytometer (FACSCalibur, Becton, Dickinson and Company, USA). Data was analyzed with FlowJo analysis software.

### RNA sequencing analysis

The 786-O cells were transfected with siNC or siOTUD1 for 48 h. These cells were subjected to RNA sequencing analysis as previously reported 16. The RNA sequencing analysis was performed by Novogene (Beijing, China). Briefly, a total of 1 μg of RNA per sample was used as the starting material for RNA. sequencing (RNA-seq). Sequencing libraries were generated using the NEBNext Ultra RNA Library Prep Kit for Illumina (NEB, USA) following the manufacturer's instructions, and index codes were added to attribute sequences to each sample. Clustering of the samples was performed on the cBot Cluster Generation System using the TruSeq PE Cluster Kit v3-cBot-HS (Illumina) according to the manufacturer's instructions. After cluster generation, libraries were sequenced on an Illumina Novaseq platform, and 150 bp paired-end reads were generated. FeatureCounts v1.5.0-p3 was used to count the read numbers mapped to each gene. Differential expression analysis was performed using the DESeq2 R package (1.16.1), and the clusterProfiler R package was used to test the statistical enrichment of differentially expressed genes (DEGs) in KEGG (Kyoto Encyclopedia of Genes and Genomes) pathways.

### Statistical analysis

Data were expressed as means ± SD. Statistical significance was determined by one-way or two-way ANOVA using GRAPHPAD PRISM 5 software, San Diego, CA, USA. A statistical significance threshold of P-values < 0.05 was used.

Other methods were provided in the supplementary information.

## Results

### OTUD1 is downregulated and correlated with an unfavorable prognosis in ccRCC patients

**Figure 1:**
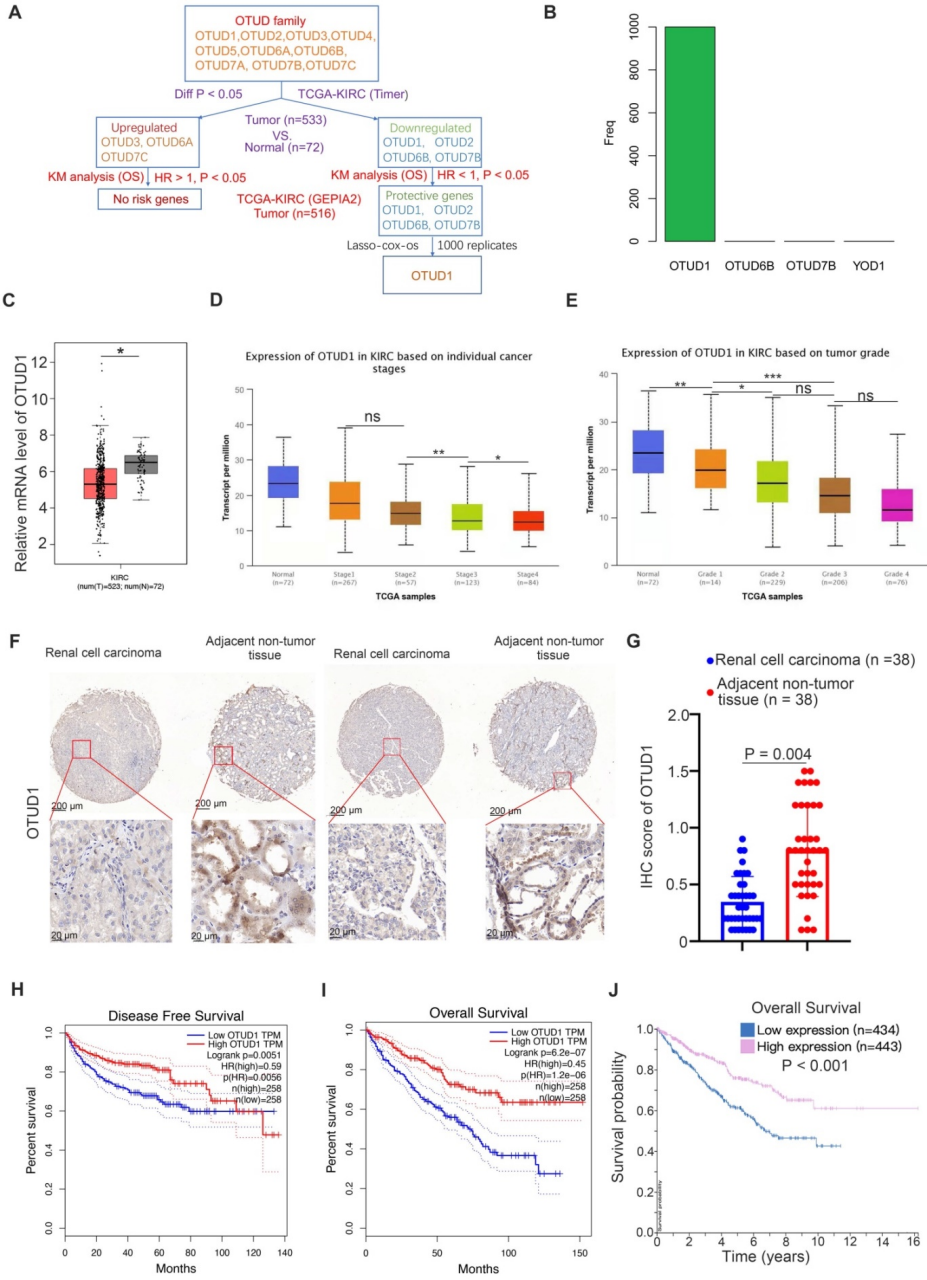
** OTUD1 is downregulated and correlated with an unfavorable prognosis in ccRCC patients. A,** Identification of OTUD1 from OTUD family in predicting prognosis of patients with KIRC. **B**, 10-fold cross-validation with 1000 replications for variable selection in the LASSO-COX-OS model by minimum criteria (the 1-SE criteria). **C**, differential expression analyses of OTUD1 between tumor and normal tissues in TCGA-KIRC dataset. *, P < 0.05. **D**, differential expression analyses of OTUD1 in the different tumor stages of renal cancer in TCGA-KIRC. ns, not significant; *, P < 0.05; **, P < 0.01. **E**, differential expression analyses of OTUD1 in the different tumor grades of renal cancer in TCGA-KIRC. ns, not significant; *, P < 0.05; **, P < 0.01. **F and G**, IHC analysis of the tissue microarray by staining the OTUD1 antibody. The typical image and expression level of OTUD1 in the non-tumor tissue and bladder cancer tissue were shown (G). P values as indicated. **H-J**, Kaplan-Meier analysis with two-sided log-rank test was conducted using GEPIA2 or The human protein atlas to evaluate the differences in Disease-free survival (H) and overall survival (I and J) time between the patients with high and low expression of OTUD1 in TCGA-KIRC dataset.

First, we studied the clinicopathological role of OTUD family members in ccRCC by analyzing The Cancer Genome Atlas (TCGA) kidney renal clear cell carcinoma (KIRC) dataset. We found that OTUD3, OTUD6A and OTUD7C were upregulated, but OTUD1, OTUD2, OTUD6B and OTUD7B were downregulated in KIRC tissues compared to normal renal tissues (Fig. 1A). Among the upregulated OTUD family members, there were no risk-related genes with hazard ratios (HRs) greater than 1 and P values less than 0.05 (Fig. 1A). Notably, there were four protective genes (HR < 1, P < 0.05) in the OTUD family, namely, OTUD1, OTUD2, OTUD6B, and OTUD7B (Fig. 1A). Then, least absolute shrinkage and selection operator (LASSO)-Cox regression analysis with 1000 replications was performed for these 4 prognostic genes in the TCGA-KIRC dataset and further showed that OTUD1 may be the key gene related to the overall survival (OS) of KIRC patients (Fig. 1B). Moreover, we showed that OTUD1 was not only downregulated in the KIRC tumor tissues but was also decreased along with increased KIRC tumor stage and grade (Fig. 1C, D, E). IHC analysis of OTUD1 in the KIRC tissue microarray (KIRC tumor n=38, nontumor tissue n=38) also showed that OTUD1 was downregulated in the tumor tissues (P =0.004) (Fig. 1F, G). Finally, we revealed that low expression of OTUD1 was associated with shorter disease-free survival and OS times in ccRCC (Fig. 1H-J). Together, these data indicated that low expression of OTUD1 is correlated with a poor prognosis in ccRCC.

### OTUD1 loss promotes cell proliferation and regulates the cell cycle of renal cancer cells

**Figure 2:**
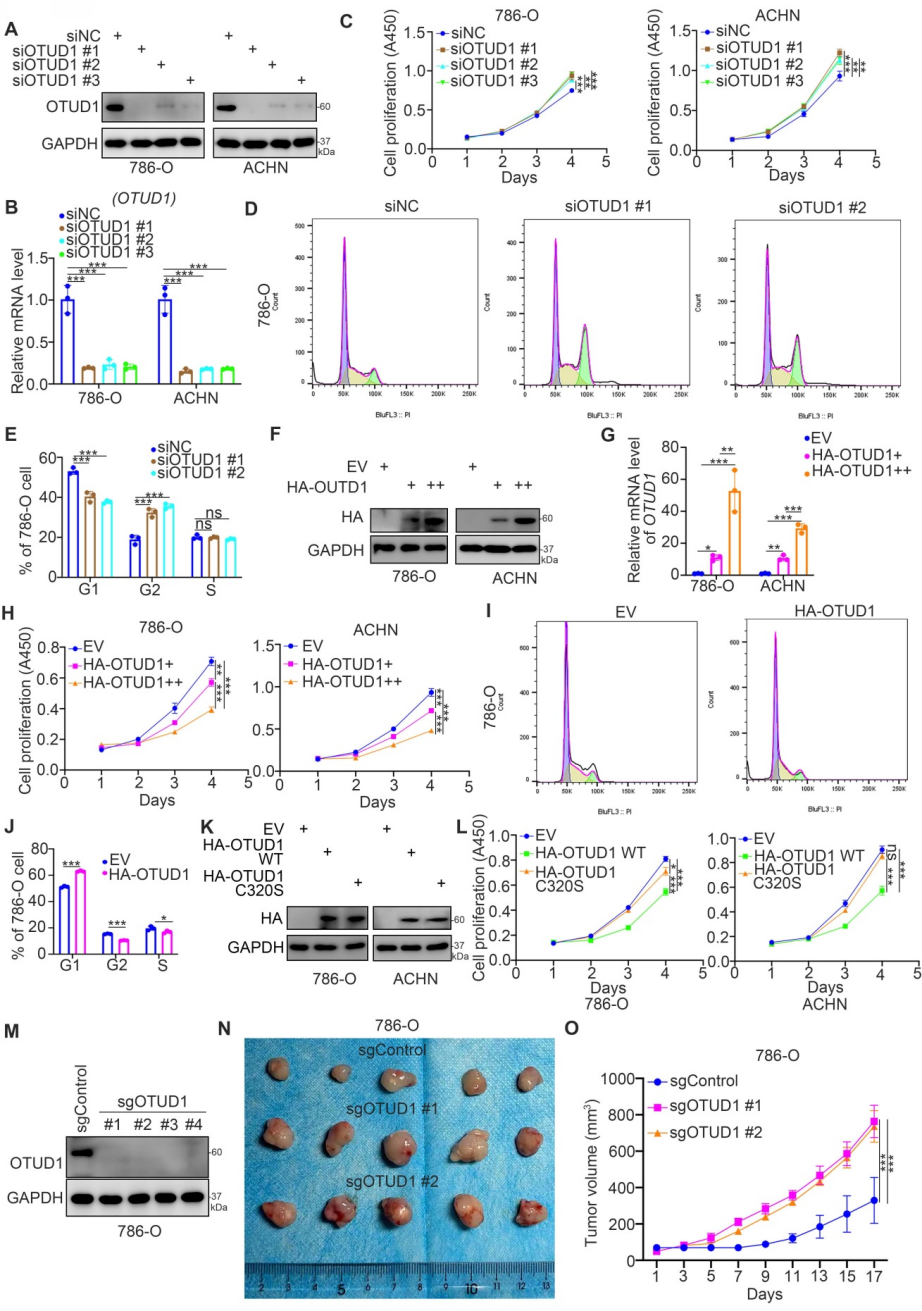
** OTUD1 loss promotes cell proliferation and regulates the cell cycle of renal cancer cells. A-E,** 786-O and ACHN cells were transfected with indicated siRNAs for 48 h. Cells were collected for Western blot analysis (A), RT-qPCR analysis (B), CCK-8 assay (C), cell cycle assay (D and E). For panel B and C, data presents as mean ± SD with three replicates. **, P < 0.01; ***, P < 0.001. For panel E, data presents as mean ± SD with two replicates. Ns, not significant; **, P < 0.01; ***, P < 0.001. **F-J**, 786-O and ACHN cells were transfected with indicated empty vector, HA-OTUD1 (2ng), or HA-OTUD1 (4ng) for 48 h. Cells were harvested for Western blot analysis (F), RT-qPCR analysis (G), CCK-8 assay (H), cell cycle assay (I and J). For panel G and H, data presents as mean ± SD with three replicates. **, P < 0.01; ***, P < 0.001. For panel J, data presents as mean ± SD with two replicates. Ns, not significant; **, P < 0.01; ***, P < 0.001. **K and L**, 786-O and ACHN cells were transfected with indicated empty vector for 48 h. Cells were harvested for Western blot analysis (K) and CCK-8 assay (L). Data presents as mean ± SD with three replicates. Ns, not significant; *, P < 0.05; ***, P < 0.001. **M-O**, 786-O cells were infected with indicated sgRNAs. After puromycin selection, cells were harvested for Western blot analysis (M). Then, cells were subcutaneously injected into the flank of nude mice. The tumor image was shown in panel N. The tumor growth curve was shown in panel O. Data presents as mean ± SD with five replicates. ***, P < 0.001.

Then, we studied the biological role of OTUD1 in renal cancer cells. The expression level of OTUD1 was downregulated after transfection with OTUD1-specific siRNAs (Fig. 2A, 2B). The cell proliferation activity of these cells was measured by Cell Counting Kit-8 (CCK-8) assay (Fig. 2C). We showed that knockdown of OTUD1 promoted cancer cell growth in both 786-O and ACHN cells (Fig. 2C). Intriguingly, cell cycle analysis indicated that OTUD1 silencing induced G1-G2 phase transition in 786-O cells (Fig. 2D, 2E). In contrast, the HA-OTUD1 constructs were transfected into 786-O and ACHN cells to ectopically overexpress OTUD1 (Fig. 2F, 2G). The CCK-8 assay showed that overexpression of OTUD1 decreased the proliferation of renal cancer cells (Fig. 2H). Moreover, a cell cycle assay indicated that overexpression of OTUD1 blocked the G1 to G2 phase transition in 786-O cells (Fig. 2I, 2J). Given that OTUD1 functioned as a deubiquitinating enzyme, we would like to investigate whether OTUD1 inhibit the proliferation of renal cancer cells through its deubiquitinating activity. The inactive mutant of OTUD1 (OTUD1 C320S)^17^, ^18^ and OTUD1 wild type (WT) were transfected into the 786-O and ACHN cells, respectively (Fig. 2K). It was not surprising that overexpression of inactive mutant of OTUD1 made little effect on the renal cancer cells proliferation compared to the OTUD1 wild type (Fig. 2L). Furthermore, the nude mouse studies showed that OTUD1 depletion led to tumor growth *in vivo* (Fig. 2M-2O). Together, these results suggest that OTUD1 contributes to inhibiting renal cancer cell proliferation.

### OTUD1 is a negative regulator of the PI3K/AKT and TNF-alpha/NF-kappa B signaling pathways in ccRCC

**Figure 3:**
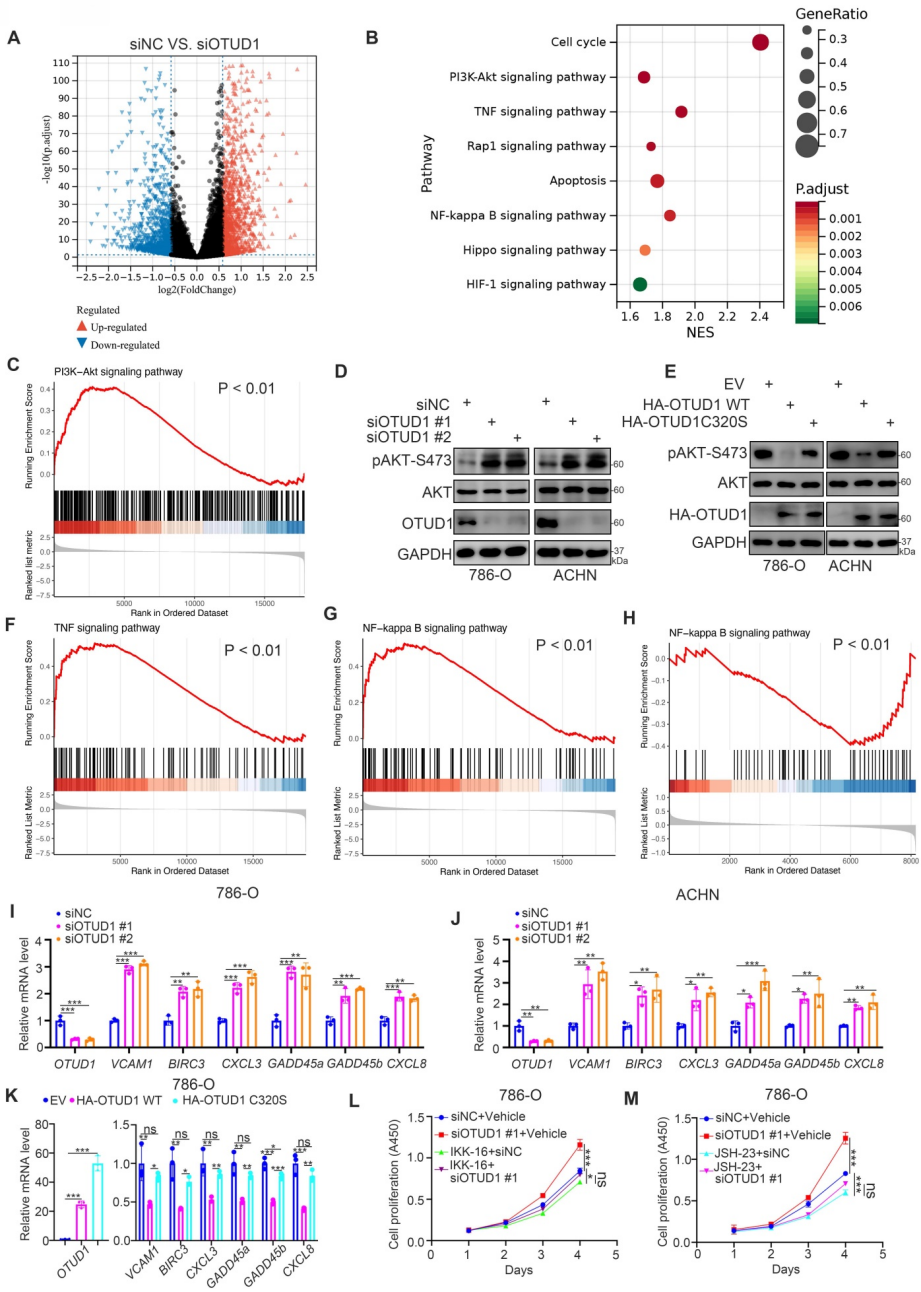
** OTUD1 is a negative regulator of the PI3K/AKT and TNF-alpha/NF-kappa B signaling pathways in ccRCC. A,** 786-O cell was transfected with siNC or siOTUD1 for 48 h. These cells were subjected to RNA-seq analysis. The volcano plot showed the genes changed after knockdown of OTUD1 (A). **B**, KEGG pathway enrichment analysis indicated that the pathways were changed after knockdown of OTUD1 in 786-O cells. **C**, GSEA analysis showed PI3K-AKT pathway was activated by knocking down OTUD1 in 786-O cells. P values as indicated. **D**, 786-O and ACHN cells were transfected with indicated siRNAs for 48 h. Cells were collected for Western blot analysis. **E**, 786-O and ACHN cells were transfected with indicated plasmids for 48 h. Cells were collected for Western blot analysis. **F and G**, GSEA analysis showed TNF signaling pathway (F) and NF-kappa B signaling (G) were activated by knocking down OTUD1 in 786-O cells. P values as indicated. **H,** NF-kappa B pathway was down-regulated in patients with high OTUD1 compared with patients with low OTUD1 expression in TCGA database.** I and J**, 786-O and ACHN cells were transfected with indicated siRNAs for 48 h. Cells were harvested for RT-qPCR analysis. Data presents as mean ± SD with three replicates. *, P < 0,05; **, P < 0.01; ***, P < 0.001. **K**, 786-O cells were transfected with indicated plasmids for 48 h. Cells were harvested for RT-qPCR analysis. Data presents as mean ± SD with three replicates. *, P < 0,05; **, P < 0.01; ***, P < 0.001. **L and M**, 786-O cells were transfected with indicated siRNAs for 24 h. These cells were treated with or without 500 nM IKK-16 (L) or 10 μM JSH-23 (M) and subjected to CCK-8 assay. Data presents as mean ± SD with three replicates. Ns, not significant; *, P < 0,05; ***, P < 0.001.

To identify the underlying mechanism by which OTUD1 inhibits ccRCC tumor growth, transcriptome sequencing after OTUD1 ablation was performed in ACHN cells (Fig. 3A, B). Kyoto Encyclopedia of Genes and Genomes (KEGG) enrichment pathway analysis showed that knockdown of OTUD1 activated a number of cellular processes and signaling pathways with P values less than 0.01 (Fig. 3C). Among them, the PI3K-AKT, TNF and NF-kappa B signaling pathways were the most altered signaling pathways after OTUD1 silencing (Fig. 3B). Gene set enrichment analysis (GSEA) of the RNA-seq dataset showed that AKT signaling was activated in the siOTUD1 group (Fig. 3C). We also found that knockdown of OTUD1 increased the phosphorylation levels of AKT, but overexpression of OTUD1 decreased the phosphorylation levels of AKT in both 786-O and ACHN cells (Fig. 3D, E). Moreover, GSEA of the RNA-seq dataset demonstrated that OTUD1 silencing resulted in the hyperactivation of TNF and the NF-kappa B signaling pathway (Fig. 3F, G). Consistently, proteomics analysis also indicated that OTUD1 was closely associated with the inhibition NF-kappa B signaling pathway in renal cell carcinoma (Fig. 3H). Furthermore, we found that OTUD1 knockdown increased the expression of downstream target genes of TNF and the NF-kappa B signaling pathway, such as TNF, BIRC3, CXCL3, GADD45a, GADD45b, and CXCL8, in 786-O and ACHN cells (Fig. 3I, J). In contrast, ectopic overexpression of OTUD1 reduced the expression of these genes in 786-O cells (Fig. 3K). NF-kappa B signaling is essential for maintaining ccRCC cell survival 19. We investigated whether OTUD1 inhibits the tumor growth of ccRCC by inactivating NF-kappa B. We treated 786-O cells with two distinct NF-kappa B inhibitors (IKK-16 and JSH-23) after silencing OTUD1 (Fig. 3L, M) and found that NF-kappa B inhibitors diminished the growth-promoting effect induced by the knockdown of OTUD1 (Fig. 3L, M). Thus, these data suggest that OTUD1 participates in inactivating the AKT and NF-kappa B/TNF-alpha signaling pathways in ccRCC.

### OTUD1 is responsible for stabilizing PTEN in ccRCC

**Figure 4:**
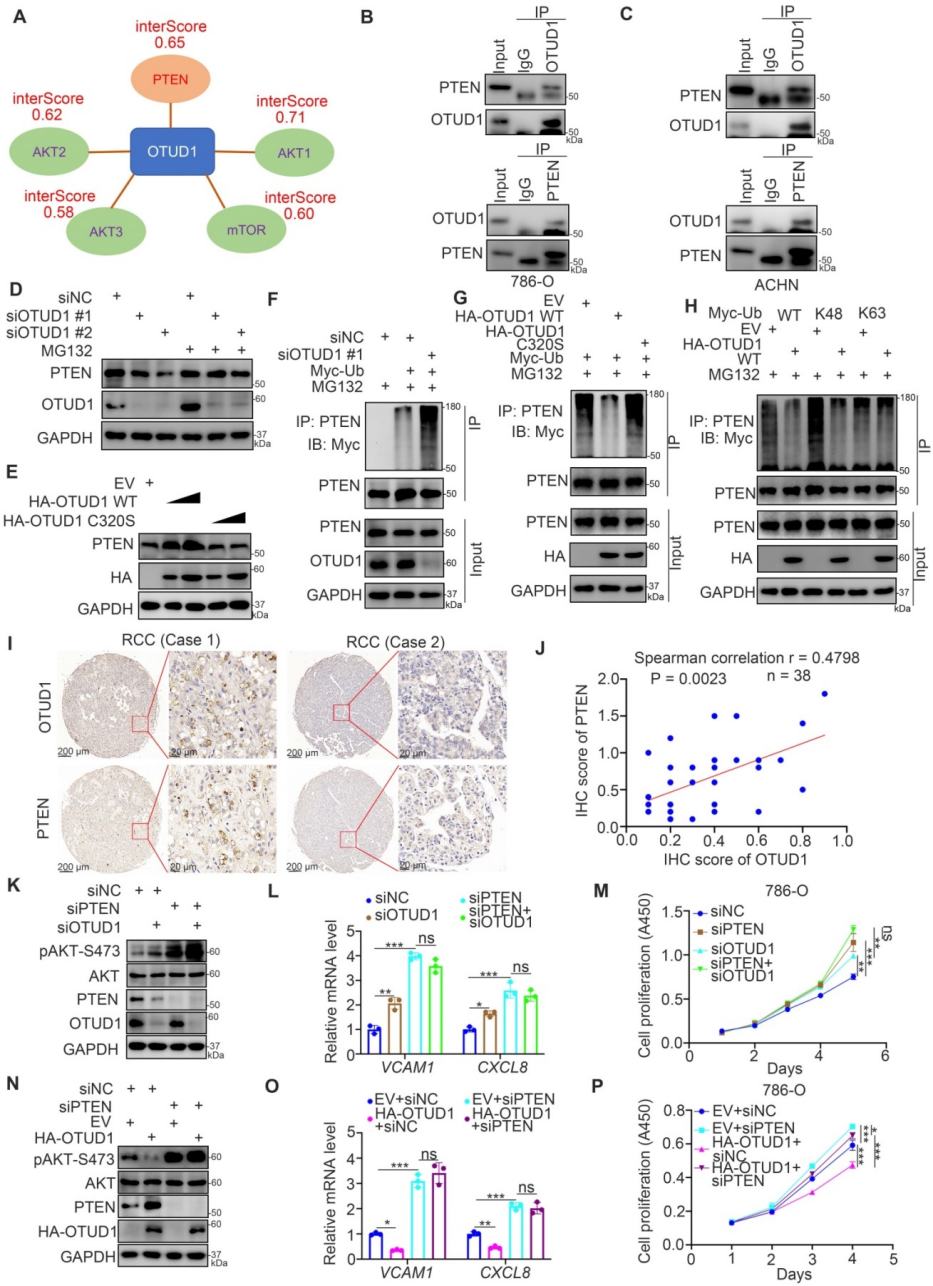
** OTUD1 is responsible for stabilizing PTEN in ccRCC. A,** We downloaded the dataset from the UbiBrowser and analyzed the interaction scores of AKT pathway related proteins with OTUD1. The interaction scores (interScore) of PTEN, AKT1, AKT2, AKT3 and mTOR were indicated in panel A. **B**, The cell lysate of 786-O cells were collected and incubated with OTUD1 or PTEN antibodies for immunoprecipitation assay. **C**, The cell lysate of ACHN cells were collected and incubated with OTUD1 or PTEN antibodies for immunoprecipitation assay. **D**, 786-O cells were transfected with indicated siRNAs for 48 h. Before harvesting cells for Western blot analysis, cells were treated with or without MG132 for 8 h.** E**, 786-O cells were transfected with indicated plasmids for 48 h. Cells were collected for Western blot analysis. **F**, 786-O cells were transfected with indicated constructs for 48 h. Before harvesting cells for immunoprecipitation by using the PTEN antibody, cells were treated with or without MG132 for 8 h. **G**, 786-O cells were transfected with indicated constructs for 48 h. Before harvesting cells for immunoprecipitation by using the PTEN antibody, cells were treated with or without MG132 for 8 h.** H,** 786-O cells were transfected with indicated constructs for 48 h. Before harvesting cells for immunoprecipitation by using the PTEN antibody, cells were treated with or without MG132 for 8 h. **I and J**, IHC analysis of the tissue microarray by staining the OTUD1 and PTEN antibodies. The correlation between these two proteins was shown in panel I, P values as indicated. **K-M**, 786-O cells were transfected with indicated siRNAs for 48 h. Cells were harvested for Western blot analysis (J), RT-qPCR analysis (K), and CCK-8 assay (L). For panel K and L, data presents as mean ± SD with three replicates. *, P < 0,05; **, P < 0.01; ***, P < 0.001. **N-P**, 786-O cells were transfected with indicated plasmids for 48 h. Cells were harvested for Western blot analysis (M), RT-qPCR analysis (N), and CCK-8 assay (O). For panel N and O, data presents as mean ± SD with three replicates. *, P < 0,05; **, P < 0.01; ***, P < 0.001.

Since OTUD1 is reported to be a deubiquitinase and involved in protein posttranslational modification, we explored the potential binding partners of OTUD1 by employing bioinformatic analysis. OTUD1 was responsible for repressing the PI3K/AKT signaling pathway and NF-kappa B signaling pathway in renal cancer cells (Fig. 3). And PI3K/AKT pathway is also reported to activate NF-kappa B signaling pathway in cells 13, 20. We downloaded the dataset from the UbiBrowser and analyzed the interaction scores of AKT pathway related proteins with OTUD1. The data was indicated in Table S3. We found that OTUD1 might interact with PTEN, AKT1, AKT2, AKT3, and mTOR among these related proteins (Fig. 4A). Given that OTUD1 promotes the activation of AKT and the NF-kappa B/TNF-alpha signaling pathway in ccRCC, OTUD1 can usually stabilize its substrates through its deubiquitinase activity. Thus, we investigated whether OTUD1 binds with and stabilized PTEN, which is a well-known negative regulator of the AKT and NF-kappa B/TNF-alpha signaling pathways 13. First, the reciprocal immunoprecipitation assay indicated that endogenous OTUD1 interacts with PTEN in 786-O and ACHN cells (Fig. 4B, C). Meanwhile, we also found that AKT did not bind with OTUD1 in 786-O cells (Supplementary Fig. 1A). Knockdown of OTUD1 decreased PTEN expression, and this process was suppressed by 26S proteasome inhibitor (MG132) treatment in 786-O cells (Fig. 4D). In contrast, overexpression of OTUD1 WT but not the catalytic inactive mutant of OTUD1 (OTUD1 C320S) led to an increase in PTEN expression in 786-O cells (Fig. 4E). Moreover, we showed that OTUD1 silencing promoted the polyubiquitination of PTEN, but OTUD1 WT overexpression suppressed the polyubiquitination of PTEN in 786-O cells (Fig. 4F, G). Notably, we also showed that the OTUD1 C320S mutant made little effect on the change of polyubiquitination of PTEN compared to OTUD1 WT (Fig. 4G). It is known that OTUD1 efficiently degrades the K63-linked ubiquitin chain 21, which does not contribute to proteasome degradation. While, Zhengkui Zhang et al. reported that OTUD1 regulates the K48-linked ubiquitination of SMAD7 to stabilize SMAD7 in breast cancer 22. We then demonstrated that OTUD1 regulates the K48-linked ubiquitination of PTEN, which suggested that OTUD1 might prevent PTEN proteasomal degradation in renal cancer cells (Fig. 4H). In addition, IHC was performed on the KIRC tissue microarray by using the PTEN antibody (Fig. 4I). We found that there was a positive correlation between OTUD1 and PTEN in KIRC tissue samples (Spearman correlation r = 0.4798, P = 0.0023) (Fig. 4J). Therefore, OTUD1 might be the deubiquitinase of PTEN in ccRCC.

Next, we wondered whether OTUD1 inhibits cancer cell growth through PTEN in ccRCC cells. 786-O cells were transfected with siNC, siPTEN, siOTUD1, or siPTEN plus siOTUD1 (Fig. 4K). We found that cotransfection with siPTEN and siOTUD1 attenuated the increase in phosphorylated AKT, VCAM1 and CXCL8 and cancer cell proliferation induced by knockdown of OTUD1 alone (Fig. 4K-M). We also showed that PTEN ablation could attenuate the decrease in phosphorylated AKT, VCAM1 and CXCL8 and cancer cell proliferation mediated by overexpression of OTUD1 in 786-O cells (Fig. 4N-P). Together, our data suggest that OTUD1 deubiquitinates and stabilizes PTEN in ccRCC.

### OTUD1 contributes to regulating the sensitivity of renal cancer cells to TKIs

**Figure 5:**
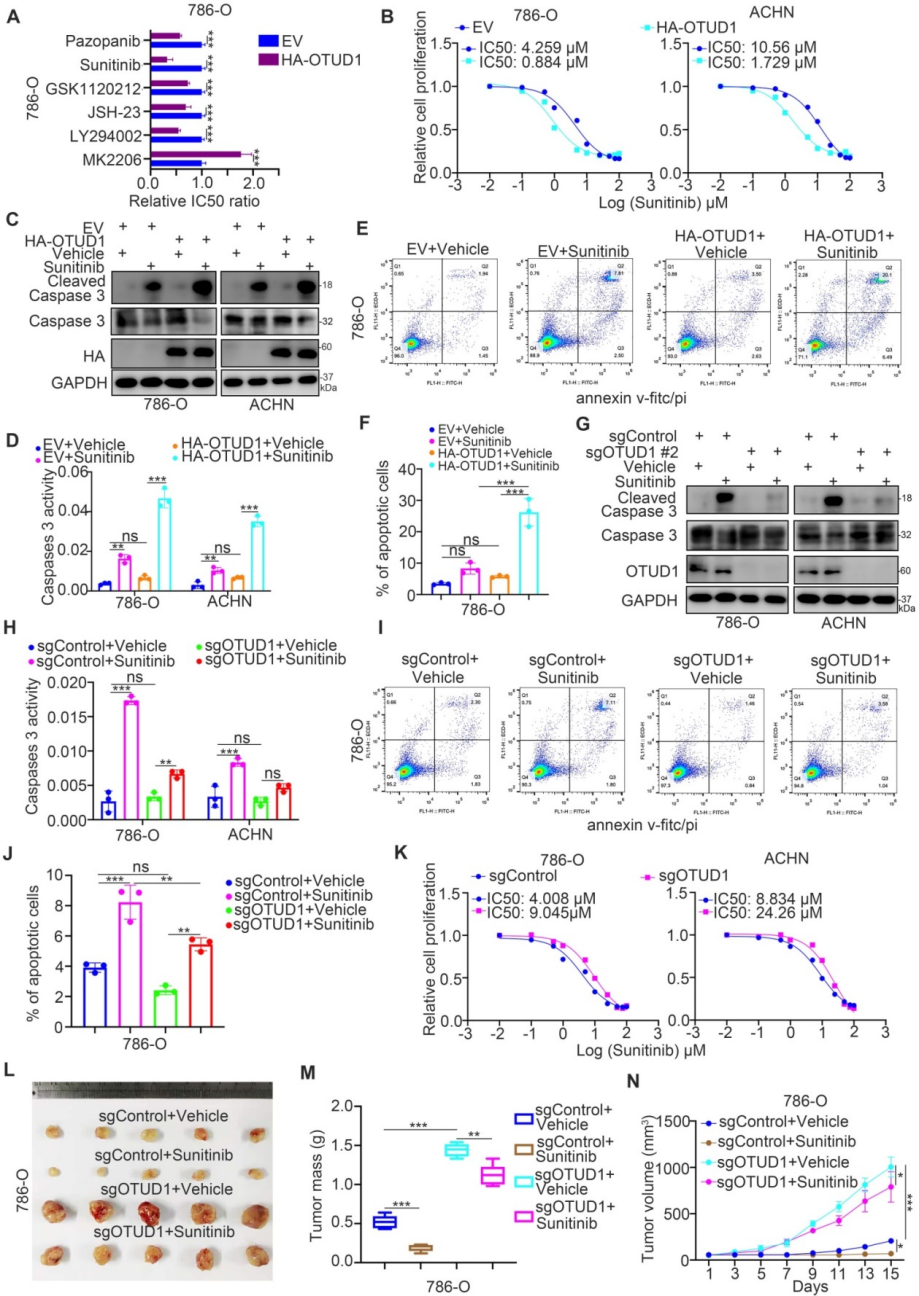
** OTUD1 contributes to regulating the sensitivity of renal cancer cells to TKIs. A,** 786-O cells were transfected indicated plasmids for 48 h. These cells were treated with a serial dose of small molecules indicated in the figure and measure the IC50 values of each small molecules. The EV/HA-OTUD1 IC50 ratios of each small molecules were shown in the panel A. Data presents as mean ± SD with three replicates. ***, P < 0.001. **B**, 786-O and ACHN cells were transfected with indicated plasmids for 48 h. Cells were treated with a serial concentration of Sunitinib for measuring the IC50 values of Sunitinib. **C-F**, 786-O and ACHN cells were transfected with indicated constructs for 48 h. Cells were treated with or without Sunitinib and collected for Western blot analysis (C), Caspase 3 activity assay (D), and Annexin v-FITC/PI assay (E and F). For panel D, E, and F, data presents as mean ± SD with three replicates. Ns, not significant; **, P < 0.01; ***, P < 0.001. **G-J,** 786-O and ACHN cells were infected with indicated constructs for 72 h. After puromycin selection, cells were treated with or without Sunitinib and collected for Western blot analysis (G), Caspase 3 activity assay (H), and Annexin v-FITC/PI assay (I and J). For panel H, I, and J, data presents as mean ± SD with three replicates. Ns, not significant; **, P < 0.01; ***, P < 0.001. **K**, 786-O and ACHN cells were infected with indicated constructs for 72 h. After puromycin selection, cells were treated a serial concentration of Sunitinib for measuring the IC50 values of Sunitinib. **L-N**, 786-O cells were infected with indicated constructs for 72 h. After puromycin selection, cells were subcutaneously injected into the flank of nude mice. Mice were treated with or without Sunitinib. The tumor image was shown in panel L. The tumor mass was shown in panel M. The tumor-growth curve was shown in panel N. Data presents as mean ± SD with five replicates. *, P <0.05; **, P < 0.01; ***, P < 0.001.

PTEN is responsible for influencing the sensitivity of cancer cells to multiple types of small molecules, such as AKT inhibitors, MEK inhibitors, and TKIs 23-25. According to the above findings, we aimed to determine whether OTUD1 regulates the sensitivity of renal cancer cells to antitumor regimens. First, we revealed that the half-maximal inhibitory concentration (IC50) values of sunitinib in the OTUD1 overexpression group were lower than those in the control group in 786-O cells (Fig. 5A). Currently, TKIs are the first-line targeted treatment for ccRCC 26. The acquired resistance to TKIs in ccRCC has become the major obstacle for improving the survival of ccRCC patients 27. Thus, we aimed to study the specific role of OTUD1 in regulating the sensitivity of renal cancer cells to TKIs. We showed that OTUD1 WT overexpression decreased the IC50 values of sunitinib in both 786-O and ACHN cells (Fig. 5B), but ectopically overexpressed the OTUD1 C320S mutant made little effect on the IC50 values of sunitinib compared to control group (empty vector) in renal cancer cells (Supplementary fig. 1B). Overexpression of OTUD1 increased apoptosis after treatment with sunitinib versus that in the control group in both 786-O and ACHN cells (Fig. 5C-F). In contrast, we showed that OTUD1 depletion decreased the apoptosis of renal cancer cells treated with sunitinib (Fig. 5G-J) and increased the IC50 values of sunitinib in 786-O and ACHN cells (Fig. 5K). Moreover, the *in vivo* study also demonstrated that OTUD1 ablation reduced the sensitivity to sunitinib (Fig. 5L-N). Together, these data suggest that OTUD1 participates in modulating the sensitivity of renal cancer cells to TKIs.

### OTUD1 sensitizes ccRCC cells to the TKIs via PTEN

**Figure 6:**
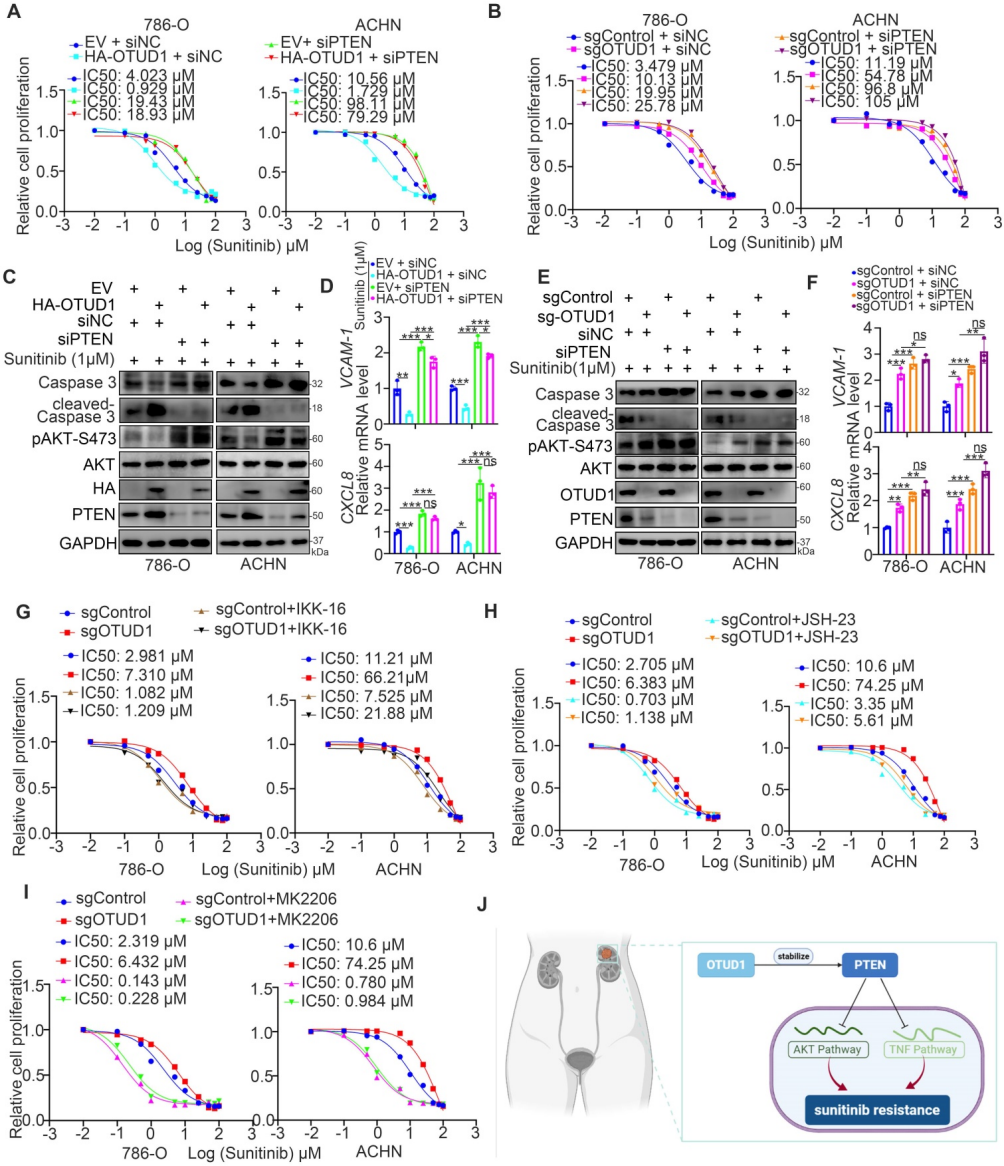
** OTUD1 sensitizes ccRCC cells to the TKIs via PTEN. A,** 786-O and ACHN cells were transfected with indicated constructs for 48 h. Cells were treated a serial concentration of Sunitinib for measuring the IC50 values of Sunitinib. **B**, 786-O and ACHN cells were infected with indicated constructs for 72 h. Cells were treated a serial concentration of Sunitinib for measuring the IC50 values of Sunitinib. **C and D,** 786-O and ACHN cells were transfected with indicated constructs for 48 h. Cells were treated with 1μM Sunitinib for another 24 h and cells were harvested for Western blot (C) and RT-qPCR analysis (D). Data presents as mean ± SD with three replicates. Ns, not significant; *, P < 0,05; **, P < 0.01; ***, P < 0.001. **E and F**, 786-O and ACHN cells were infected with indicated constructs for 72 h. Cells were treated with 1μM Sunitinib for another 24 h and cells were harvested for Western blot (E) and RT-qPCR analysis (F). Data presents as mean ± SD with three replicates. Ns, not significant; *, P < 0,05; **, P < 0.01; ***, P < 0.001. **G**, 786-O and ACHN cells were infected with indicated constructs for 72 h. Then, these cells were pretreated with or without IKK-16 (500 nM) and treated a serial concentration of Sunitinib for measuring the IC50 values of Sunitinib. **H**, 786-O and ACHN cells were infected with indicated constructs for 72 h. Then, these cells were pretreated with or without JSH-23 (10 μM) and treated a serial concentration of Sunitinib for measuring the IC50 values of Sunitinib. **I**, 786-O and ACHN cells were infected with indicated constructs for 72 h. Then, these cells were pretreated with or without MK2206 (10 μM) and treated a serial concentration of Sunitinib for measuring the IC50 values of Sunitinib. **J**, a model depicting that the downregulation of OTUD1 stabilizes PTEN to inactivate AKT and TNF/NF-kappa B pathway and be responsible for the Sunitinib resistance in ccRCC.

Next, we investigated whether OTUD1 regulates the sensitivity of renal cancer cells to TKIs through PTEN. It was not surprising that PTEN silencing diminished the change in IC50 values of sunitinib, AKT phosphorylation, VCAM-1, CXCL8 and cleaved caspase 3 levels induced by silencing or overexpressing OTUD1 in ccRCC cells (Fig. 6A-F). We previously analyzed a TKI resistance dataset (GSE76068) from a mouse model and showed that RRM2 is closely associated with sunitinib resistance in renal cancer, and this effect is mediated by its activation of the AKT signaling pathway 28. It has been also documented that the NF-kappa B signaling pathway was involved in modulating the sensitivity of TKIs in renal cancer cells 29. In consist with previously finding, we reanalyzed the TKI resistance dataset (GSE76068) and performed KEGG pathway enrichment analysis to show that TNF and NF-kappa B signaling pathways were correlated with TKI resistance (Supplementary fig. 1C-E). Moreover, we showed that inhibition of the TNF/NF-kappa B signaling pathway enhanced the antitumor effect of TKIs in both 786-O and ACHN cells (Supplementary fig. 1F-I).

OTUD1 plays a key role in modulating the activation of AKT and TNF/NF-kappa B signaling in ccRCC. PTEN acts as a negative regulator of the AKT and TNF/NF-kappa B signaling pathways 13, and abnormal activation of AKT and TNF/NF-kappa B signaling contributes to TKI resistance in ccRCC 30, 31. Thus, we aimed to study whether the effect of OTUD1 on the sensitivity of renal cancer cells to TKIs is mediated by the AKT and TNF/NF-kappa B signaling pathways. 786-O and ACHN cells were transfected with sgControl or sgOTUD1 and pretreated with TNF/NF-kappa B pathway and AKT pathway inhibitors (Fig. 6G-I). Then, the IC50 values of sunitinib were assessed. We demonstrated that IKK-16, JSH-23, and MK2206 attenuated the increase in the IC50 of sunitinib induced by OTUD1 ablation in both 786-O and ACHN cells (Fig. 6G-I). Thus, our results suggest that OTUD1 is involved in modulating the sensitivity of ccRCC to TKIs via PTEN (Fig. 6J).

## Discussion

Studies have mentioned that OTUD1 plays a key role in modulating the immune response to viral infection. For instance, OTUD1 increases Smurf1 expression levels to promote degradation of the MAVS/TRAF3/TRAF6 signalosome and inhibit the innate immune response to viral infection 32. OTUD1 deubiquitinates CARD9 to modulate antifungal innate immunity 33. Meanwhile, OTUD1 is lost in multiple types of human cancer 22 and closely associated with cell survival and apoptosis. It has been well documented that OTUD1 deubiquitinates AIF at K244 to disrupt the mitochondrial structure and deubiquitinates AIF at K255 to promote its DNA-binding activity, which activates the caspase-independent apoptotic pathway 34. On the other hand, OTUD1 increases the stability of DCAF10 and DDB1 to activate the caspase-independent apoptotic pathway in cells 34. However, Wu et al. reported that OTUD1 stabilizes MCL1 to block BH3-mimetic inhibitor-induced cell death in some types of cancer, including ovarian cancer, liver cancer and cervical cancer 35. Moreover, Zhang et al. reported that OTUD1 binds with and stabilizes SMAD7 to repress the metastasis of breast cancer 22. Here, we showed that low expression of OTUD1 is involved in the activation of the PI3K/AKT and TNF-alpha/NF-kappa B signaling pathways by decreasing the stability of PTEN in ccRCC cells. Interestingly, OTUD3, another OTUD family member, regulates the stability of PTEN and repress tumorigenesis 36. These findings indicated that OTUD1 and OTUD3 might share similar binding sites with PTEN. The mechanism of OTUD1 in regulating cancer progression is still unclear and needs to be further studied.

TKIs have been proven to be a promising therapeutic strategy and are currently recognized as the first-line treatment for ccRCC 37. With the prolongation of the survival time of patients with ccRCC, acquired resistance to TKIs is the major factor determining the OS time of these patients 38. Studies have reported that sunitinib treatment activates many resistance-associated pathways in renal cancer cells, such as the pERK-induced endoplasmic reticulum stress response pathway, PI3K/AKT signaling pathway, and NF-kappa B pathway 29. The pERK-induced endoplasmic reticulum stress response upregulates the expression of IL-6, IL-8, and TNF-α 29. Blockade of pERK or administration of IL-6 neutralizing antibody or NF-kappa B inhibitors could restore the sensitivity of renal cancer cells to sunitinib 29, 31. In this study, we reanalyzed a TKI resistance dataset (GSE76068) and found that TNF and the NF-kappa B signaling pathway are very closely associated with sunitinib resistance in renal cancer. Treatment with TNF and NF-kappa B inhibitors sensitized renal cancer cells to sunitinib. Thus, the NF-kappa B pathway is an attractive therapeutic target for overcoming sunitinib resistance in ccRCC 19. The AKT signaling pathway is also important for modulating the sensitivity of renal cancer cells to TKIs, and combination with AKT inhibitor treatment improved the anticancer effect of TKIs in ccRCC 28, 39. Here, we showed that OTUD1 loss triggers the AKT and NF-kappa B signaling pathways and promotes TKI resistance partially by stabilizing PTEN in ccRCC. However, we believe that there must be other molecular mechanisms regulating TKI resistance mediated by OTUD1. Therefore, further studies are needed.

Collectively, we demonstrated that OTUD1 is downregulated in renal cancer and involved in the poor prognosis of renal cancer. Then, we showed that OTUD1 inhibits cancer cell growth. Moreover, analysis of OTUD1 RNA-seq data indicated that OTUD1 inhibition triggers the AKT and NF-kappa B pathways in renal cancer cells. Furthermore, OTUD1 interacts with PTEN and regulates its stability. Subsequently, we revealed that downregulation of OTUD1 contributes to the sensitivity of renal cancer cells to TKIs, and this effect was blocked by TNF/NF-kappa B inhibitors and AKT inhibitors. Thus, we identified that the OTUD1-PTEN axis suppresses tumor growth and regulates the resistance of renal cancer to TKIs.

## Supplementary Material

Supplementary methods, figure and tables.

## Abbreviations

OTUD1: OTU domain-containing protein 1
PTEN: Phosphatidylinositol 3,4,5-trisphosphate 3-phosphatase and dual-specificity protein phosphatase
TKI: Tyrosine kinase inhibitor
RCC: Renal cell carcinoma
ccRCC: Clear cell renal cell carcinoma
TCGA: The Cancer Genome Atlas
KIRC: Kidney renal clear cell carcinoma
