# Supplementary material for: OTUD1 stabilizes PTEN to inhibit the PI3K/AKT and TNF-alpha/NF-kappaB signaling pathways and sensitize ccRCC to TKIs
Source: Int J Biol Sci. 2022 Jan 24;18(4):1401–14. doi: 10.7150/ijbs.68980 (PMC8898358; doi:10.7150/ijbs.68980)
Supplement: Supplementary file 1 — Supplementary methods, figure and tables. [file ijbsv18p1401s1.pdf]

OTUD1 stabilizes PTEN to inhibit the PI3K/AKT and TNF-alpha/NF-kappaB signaling pathways and sensitize ccRCC to TKIs

Wentao Liu, Bin Yan, Haixin Yu, Jiannan Ren, Mou Peng, Liang Zhu, Yinhuai Wang, Xin Jin, Lu Yi

Supplementary figure 1

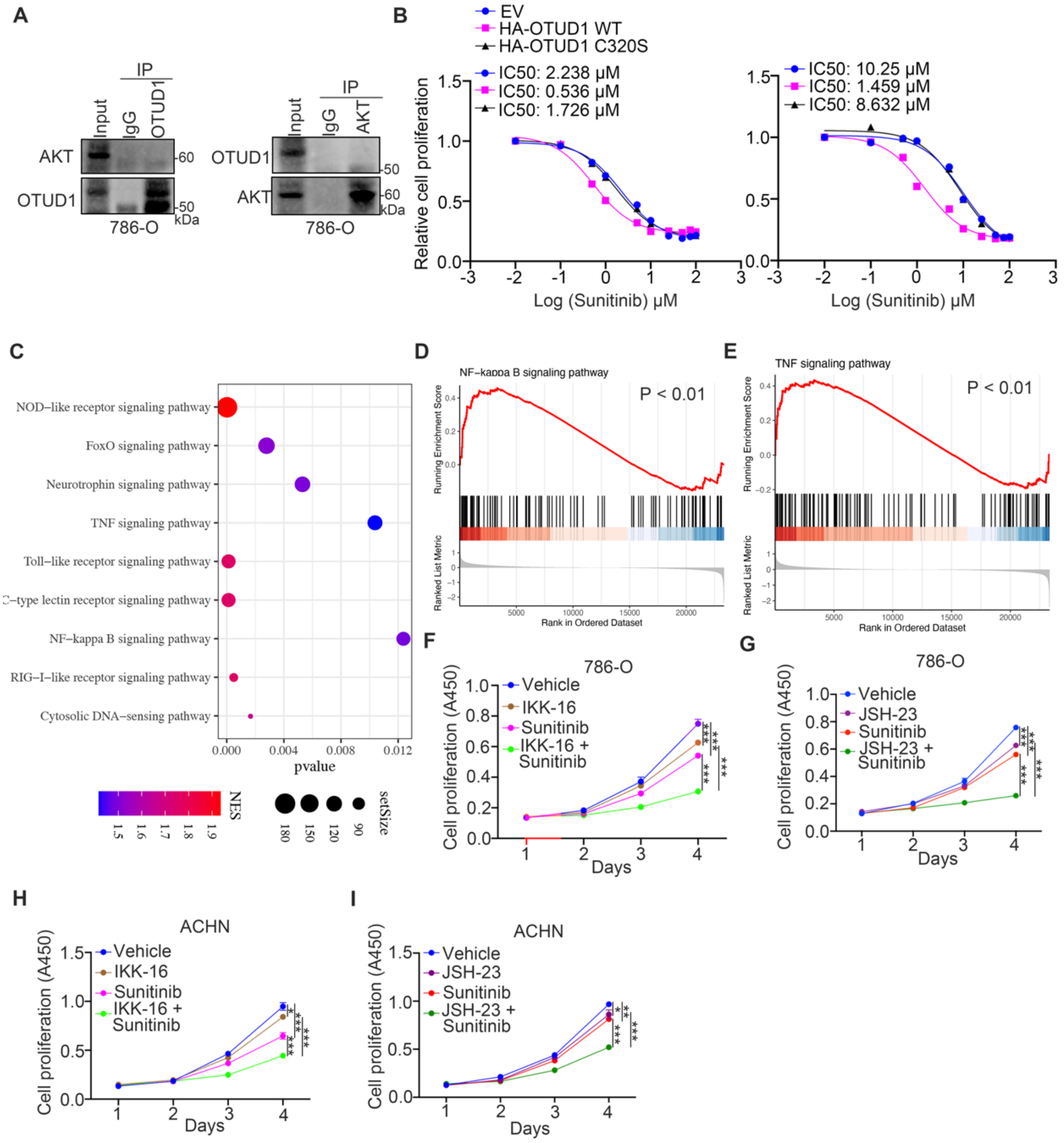

Supplementary figure 1. A, The cell lysate of 786-O cells were collected and incubated with OTUD1 or AKT antibodies for immunoprecipitation assay. **B**, 786-O and ACHN cells were transfected with indicated constructs for 48 h. Cells were treated a serial concentration of Sunitinib for measuring the IC50 values of Sunitinib. **C**, KEGG pathway enrichment analysis to show that several signaling pathway was involved in regulating the TKIs resistance in renal cancer. **D and E**, GSEA analysis showed NF-kappa B pathway and TNF signaling pathway were activated in sunitinib resistance group. P values as indicated. **F and G,** 786-O cells treated with indicated small molecules and subjected to CCK-8 assay. Data presents as mean ± SD with three replicates. ***, P < 0.001. **H and I,** ACHN cells treated with indicated small molecules and subjected to CCK-8 assay. Data presents as mean ± SD with three replicates. *, P <0.05; **, P <0.01; ***, P < 0.001.

Supplementary Material and Methods

LASSO-COX regression analysis

First, we utilized univariate Cox regression analysis to identify OTUD family genes in kidney cancer samples in comparison with adjacent kidney samples with survival data in TCGA-KIRC cohort with significant association with overall survival using the “survival” R package. Subsequentially, we used the least absolute shrinkage and selection operator (LASSO) regression analysis to further identify prognostic related genes using the R “glmnet” package. 1000 times repeated cross-validation for tuning

parameter selection was run to prevent overfitting and the partial likelihood deviance met the minimum criteria. Finally, we selected the significant gene for further analysis.

GSEA for the key gene

GSEA: KIRC patients were first divided into two groups according to the median expression level of the key gene. Then, differential expression analysis was applied between the high and low expression groups. Input genes for GSEA were sorted by their logFC values. Signaling pathways activated or suppressed by the key gene were decided by the normalized enrichment score (NES) value derived from GSEA.

ssGSEA: ssGSEA was used to calculate separate enrichment scores for each pairing of a KIRC sample and KEGG gene set. The ssGSEA score was further rescaled by min-max normalization method. Correlation analysis was performed between expression values of key gene and NES of signaling pathways.

Survival analysis

KIRC patients were divided into two groups according to the median expression level of the key gene. The differences in RFS and OS between the high and low expression groups were evaluated by the Kaplan–Meier method, followed by a log-rank test.

Table S1. The shRNA and siRNA sequences.

siNC	5'- ACGTGACACGTTCGGAGAATT-3'
siOTUD1 #1	5'- ATGCTGAATGTGAATATCCATTT-3'

siOTUD1#2	5'- CTGAATGTGAATATCCATTTAAC-3'
siOTUD1#3	5'- TCCTTGTCTAAAATGTATATTGA-3'
siPTEN #1	5'- GAGATCGTTAGCAGAAACAAAAG-3'
siPTEN #2	5'- CACAAGATGATGTTTGAAACTAT-3'
sgControl	5'- GGCTCTGCTGAACTCCAAGG-3'
sgOTUD1 #1	5'-CTCCGCCGCACTACTCGGGG-3'
sgOTUD1 #2	5'-GCCAAGTGCTCTTCGCCCCA-3'
sgOTUD1 #3	5'-AGAAGCAGGACAAGTATCTG-3'

Table S2. The primer sequences for RT-qPCR.

Gene （Human）	Forward primer (5′ - 3′)	Reverse primer (5′ - 3′)
GAPDH	ATGACAATGAATACGGCTACAGCA	GCAGCGAACTTTATTGATGGTATT
OTUD1	GCTGTCAGCAAGACGGTGTA	TAGACACCGTGGGACTCTCC
VCAM1	CAGACAGGAAGTCCCTGGAA	TTCTTGCAGCTTTGTGGATG
BIRC3	CTTTGCCTGTGGTGGAAAAT	ACTTGCAAGCTGCTCAGGAT
CXCL3	GCAGGGAATTCACCTCAAGA	GGTGCTCCCCTTGTTCAGTA
GADD45a	ACGAGGACGACGACAGAGAT	GCAGGATCCTTCCATTGAGA
GADD45b	CACCCTGATCCAGTCGTTCT	TGACAGTTCGTGACCAGGAG
CXCL8	GTGCAGTTTTGCCAAGGAGT	CTCTGCACCCAGTTTTCCTT

Table S3. The interaction scores of AKT pathway related proteins with OTUD1.

	Symbol.dub	Symbol.sub	go	interLR	interScore
1	OTUD1	LAMB4	GO:0070536 GO:0016477	2.28	0.58854043
2	OTUD1	COL6A6	GO:0070536 GO:0007155	4.28	0.65281676
3	OTUD1	COL6A5	GO:0070536 GO:0007155	1.32	0.53010702
4	OTUD1	SGK1	GO:0070536 GO:0006974	2.28	0.58854043
5	OTUD1	TLR4	GO:0070536 GO:0042088	2.6268	0.60334613
6	OTUD1	PIK3CD	GO:0070536 GO:0014065	1.32	0.53010702
7	OTUD1	PIK3R2	GO:0004843 GO:0030971	1.48	0.5424629
8	OTUD1	GNB5	GO:0070536 GO:0043547	1.32	0.53010702
9	OTUD1	IKBKB	GO:0004843 GO:0008384	4.958	0.66714635
10	OTUD1	EREG	GO:0070536 GO:0045410	2.6268	0.60334613
11	OTUD1	CHUK	GO:0004843 GO:0008384	3.3744	0.62906239
12	OTUD1	ANGPT2	GO:0004843 GO:0030971	4.958	0.66714635
13	OTUD1	LAMA5	GO:0070536 GO:0045995	4.09	0.64833381
14	OTUD1	CHAD	GO:0070536 GO:0060348	1.32	0.53010702
15	OTUD1	FGF10	GO:0070536 GO:0032925	1.32	0.53010702
16	OTUD1	PDPK1	GO:0070536 GO:0043122	1.32	0.53010702
17	OTUD1	FOXO3	GO:0070536 GO:0030330	3.35	0.62832665
18	OTUD1	CREB3	GO:0004843 GO:0035497	1.9536	0.5722007
19	OTUD1	VEGFD	GO:0070536 GO:0071542	1.32	0.53010702
20	OTUD1	PHLPP1	GO:0070536 GO:0046328	1.32	0.53010702
21	OTUD1	JAK2	GO:0070536 GO:0033209	3.35	0.62832665
22	OTUD1	ITGA10	GO:0070536 GO:0007160	1.32	0.53010702
23	OTUD1	EGFR	GO:0004843 GO:0005006	4.958	0.66714635
24	OTUD1	MYC	GO:0004843 GO:0001047	1.48	0.5424629
25	OTUD1	HRAS	GO:0070536 GO:0042088	1.99	0.57416212
26	OTUD1	PDGFB	GO:0004843 GO:0046982	1.9536	0.5722007
27	OTUD1	EGF	GO:0004843 GO:0017147	13.6604	0.75684574
28	OTUD1	TGFA	GO:0070536 GO:0050679	1.32	0.53010702
29	OTUD1	PRL	GO:0070536 GO:0030879	1.32	0.53010702
30	OTUD1	INS	GO:0070536 GO:0045861	1.99	0.57416212
31	OTUD1	IFNA2	GO:0004843 GO:0005125	1.48	0.5424629
32	OTUD1	IFNA10	GO:0004843 GO:0005125	1.48	0.5424629
33	OTUD1	IFNA7	GO:0004843 GO:0005125	1.48	0.5424629
34	OTUD1	IFNA21	GO:0004843 GO:0005125	1.48	0.5424629
35	OTUD1	IFNA5	GO:0004843 GO:0005125	1.48	0.5424629
36	OTUD1	IFNA14	GO:0004843 GO:0005125	1.48	0.5424629
37	OTUD1	IFNA17	GO:0004843 GO:0005125	1.48	0.5424629
38	OTUD1	IFNB1	GO:0004843 GO:0005125	1.9536	0.5722007
39	OTUD1	EPO	GO:0070536 GO:0009651	2.6268	0.60334613
40	OTUD1	COL1A1	GO:0070536 GO:0001503	2.28	0.58854043
41	OTUD1	COL2A1	GO:0070536 GO:2001240	2.28	0.58854043

42	OTUD1	COL4A1	GO:0070536 GO:0007528	1.32	0.53010702
43	OTUD1	FN1	GO:0004843 GO:0097718	7.5256	0.70610495
44	OTUD1	VTN	GO:0070536 GO:0010951	4.28	0.65281676
45	OTUD1	RAF1	GO:0004843 GO:0008179	1.48	0.5424629
46	OTUD1	PDGFA	GO:0004843 GO:0046982	1.48	0.5424629
47	OTUD1	VWF	GO:0004843 GO:0047485	1.9536	0.5722007
48	OTUD1	ERBB2	GO:0004843 GO:0019903	8.4064	0.7159805
49	OTUD1	NTRK1	GO:0004843 GO:0005166	1.84	0.56582025
50	OTUD1	IFNA6	GO:0004843 GO:0005125	1.48	0.5424629
51	OTUD1	IFNA4	GO:0004843 GO:0005125	1.48	0.5424629
52	OTUD1	IFNA16	GO:0004843 GO:0005125	1.48	0.5424629
53	OTUD1	ITGB3	GO:0070536 GO:0032147	1.32	0.53010702
54	OTUD1	IL4	GO:0004843 GO:0005125	3.3744	0.62906239
55	OTUD1	IL6	GO:0004843 GO:0005125	1.9536	0.5722007
56	OTUD1	ITGB1	GO:0004843 GO:1990782	1.48	0.5424629
57	OTUD1	INSR	GO:0070536 GO:0045725	1.32	0.53010702
58	OTUD1	ITGAV	GO:0004843 GO:0050431	1.9536	0.5722007
59	OTUD1	CSF1R	GO:0004843 GO:0019903	1.9536	0.5722007
60	OTUD1	HSP90AA1	GO:0004843 GO:0097718	1.84	0.56582025
61	OTUD1	LAMB1	GO:0070536 GO:0050679	9.23	0.72416206
62	OTUD1	THBS1	GO:0004843 GO:0050431	4.958	0.66714635
63	OTUD1	IGF1R	GO:0070536 GO:0046328	4.09	0.64833381
64	OTUD1	COL1A2	GO:0070536 GO:0032963	2.28	0.58854043
65	OTUD1	NGFR	GO:0004843 GO:0048406	5.626368	0.67922841
66	OTUD1	CHRM2	GO:0004843 GO:1990763	1.9536	0.5722007
67	OTUD1	HSP90AB1	GO:0070536 GO:0009651	1.99	0.57416212
68	OTUD1	ITGA2B	GO:0070536 GO:0007160	4.28	0.65281676
69	OTUD1	COL4A2	GO:0070536 GO:0071560	1.32	0.53010702
70	OTUD1	MET	GO:0004843 GO:0019903	1.9536	0.5722007
71	OTUD1	ITGA5	GO:0070536 GO:0030949	1.32	0.53010702
72	OTUD1	IL3	GO:0004843 GO:0005125	1.9536	0.5722007
73	OTUD1	FGF2	GO:0004843 GO:0005125	1.48	0.5424629
74	OTUD1	CSF1	GO:0070536 GO:0045672	2.6268	0.60334613
75	OTUD1	PDGFRB	GO:0070536 GO:2000379	1.32	0.53010702
76	OTUD1	CSF3	GO:0004843 GO:0005125	1.48	0.5424629
77	OTUD1	MYB	GO:0070536 GO:0048661	4.09	0.64833381
78	OTUD1	BCL2	GO:0004843 GO:0051721	1.48	0.5424629
79	OTUD1	SPP1	GO:0004843 GO:0005125	1.48	0.5424629
80	OTUD1	LAMC1	GO:0070536 GO:0065003	3.35	0.62832665
81	OTUD1	CHRM1	GO:0070536 GO:0007165	1.32	0.53010702
82	OTUD1	FGF3	GO:0070536 GO:0007165	5.68	0.68012544
83	OTUD1	CDK4	GO:0070536 GO:2000134	1.32	0.53010702

84	OTUD1	COL6A2	GO:0070536 GO:0009749	1.32	0.53010702
85	OTUD1	COL6A3	GO:0070536 GO:0007517	1.32	0.53010702
86	OTUD1	IL7	GO:0070536 GO:0002360	1.99	0.57416212
87	OTUD1	ITGA4	GO:0004843 GO:0046982	1.9536	0.5722007
88	OTUD1	OSM	GO:0004843 GO:0005125	1.48	0.5424629
89	OTUD1	GYS1	GO:0070536 GO:0005978	1.32	0.53010702
90	OTUD1	HGF	GO:0070536 GO:0030212	1.32	0.53010702
91	OTUD1	HSP90B1	GO:0004843 GO:0019903	1.48	0.5424629
92	OTUD1	IL2RB	GO:0070536 GO:0065003	1.32	0.53010702
93	OTUD1	ATF2	GO:0004843 GO:0035497	1.48	0.5424629
94	OTUD1	AREG	GO:0004843 GO:0005125	1.48	0.5424629
95	OTUD1	VEGFA	GO:0004843 GO:0005125	1.48	0.5424629
96	OTUD1	ITGB4	GO:0070536 GO:0006914	3.35	0.62832665
97	OTUD1	CREB1	GO:0070536 GO:0045672	2.6268	0.60334613
98	OTUD1	PDGFRA	GO:0070536 GO:0042475	3.35	0.62832665
99	OTUD1	PRLR	GO:0070536 GO:0007171	3.35	0.62832665
100	OTUD1	GNB3	GO:0004843 GO:0051020	1.9536	0.5722007
101	OTUD1	IL7R	GO:0070536 GO:0038111	1.32	0.53010702
102	OTUD1	IFNAR1	GO:0070536 GO:0009615	4.09	0.64833381
103	OTUD1	PRKCA	GO:0070536 GO:0031666	7.565184	0.70657753
104	OTUD1	ITGA2	GO:0070536 GO:0060100	4.09	0.64833381
105	OTUD1	FLT1	GO:0070536 GO:0030949	2.28	0.58854043
106	OTUD1	ITGB5	GO:0070536 GO:0007160	1.32	0.53010702
107	OTUD1	ITGB6	GO:0070536 GO:0007160	3.35	0.62832665
108	OTUD1	ATF4	GO:0004843 GO:0001085	1.48	0.5424629
109	OTUD1	EPOR	GO:0070536 GO:0046697	1.32	0.53010702
110	OTUD1	NFKB1	GO:0004843 GO:0001225	5.626368	0.67922841
111	OTUD1	EFNA1	GO:0070536 GO:0010719	1.32	0.53010702
112	OTUD1	COL9A1	GO:0070536 GO:0009887	1.32	0.53010702
113	OTUD1	KITLG	GO:0004843 GO:0005125	4.958	0.66714635
114	OTUD1	IBSP	GO:0070536 GO:0045785	3.35	0.62832665
115	OTUD1	ERBB3	GO:0004843 GO:0046982	4.958	0.66714635
116	OTUD1	TNXB	GO:0070536 GO:0032963	2.28	0.58854043
117	OTUD1	FGFR4	GO:0070536 GO:0042593	1.32	0.53010702
118	OTUD1	NR4A1	GO:0004843 GO:0046982	1.9536	0.5722007
119	OTUD1	ITGA6	GO:0070536 GO:0050873	4.09	0.64833381
120	OTUD1	RPS6KB1	GO:0070536 GO:0071363	1.32	0.53010702
121	OTUD1	JAK1	GO:0004843 GO:0019903	1.9536	0.5722007
122	OTUD1	BDNF	GO:0070536 GO:0007406	1.99	0.57416212
123	OTUD1	EIF4B		1.32	0.53010702
124	OTUD1	LAMA2	GO:0070536 GO:0045995	5.68	0.68012544
125	OTUD1	CCND1	GO:0004843 GO:0042826	1.9536	0.5722007

126	OTUD1	IL4R	GO:0070536 GO:0120162	1.32	0.53010702
127	OTUD1	TNC	GO:0070536 GO:0042475	3.35	0.62832665
128	OTUD1	CCNE1	GO:0070536 GO:1900087	2.28	0.58854043
129	OTUD1	CDK2	GO:0070536 GO:0031571	1.32	0.53010702
130	OTUD1	F2R	GO:0070536 GO:0030193	3.35	0.62832665
131	OTUD1	LAMA1	GO:0070536 GO:0045995	3.35	0.62832665
132	OTUD1	ITGA3	GO:0004843 GO:0046982	1.9536	0.5722007
133	OTUD1	ITGB7	GO:0070536 GO:0007160	1.32	0.53010702
134	OTUD1	ITGB8	GO:0070536 GO:0010628	4.28	0.65281676
135	OTUD1	IL3RA	GO:0070536 GO:0019221	1.32	0.53010702
136	OTUD1	YWHAQ	GO:0004843 GO:0047485	1.48	0.5424629
137	OTUD1	MAPK3	GO:0070536 GO:0002741	1.32	0.53010702
138	OTUD1	PIK3R1	GO:0004843 GO:0019903	3.3744	0.62906239
139	OTUD1	MAPK1	GO:0004843 GO:0031435	2.4288	0.59517287
140	OTUD1	EPHA2	GO:0070536 GO:0030316	1.32	0.53010702
141	OTUD1	COL4A5	GO:0070536 GO:0007528	3.35	0.62832665
142	OTUD1	NOS3	GO:0070536 GO:0050999	1.32	0.53010702
143	OTUD1	PPP2R1A	GO:0004843 GO:0046982	1.9536	0.5722007
144	OTUD1	PPP2R1B		1.32	0.53010702
145	OTUD1	CCND3	GO:0070536 GO:0001934	1.32	0.53010702
146	OTUD1	AKT1	GO:0070536 GO:0045861	8.1391	0.71311809
147	OTUD1	AKT2	GO:0070536 GO:0010748	3.35	0.62832665
148	OTUD1	IL2RG	GO:0070536 GO:0038111	1.32	0.53010702
149	OTUD1	YWHAB	GO:0004843 GO:0042826	1.9536	0.5722007
150	OTUD1	IFNA8	GO:0004843 GO:0005125	1.48	0.5424629
151	OTUD1	THBS2	GO:0070536 GO:0051965	3.35	0.62832665
152	OTUD1	THBS4	GO:0070536 GO:0034103	8.1391	0.71311809
153	OTUD1	PCK1	GO:0070536 GO:0006006	1.32	0.53010702
154	OTUD1	IRS1	GO:0070536 GO:0046676	3.35	0.62832665
155	OTUD1	FLT4	GO:0004843 GO:0019903	6.3344	0.69033918
156	OTUD1	KDR	GO:0070536 GO:0002042	3.35	0.62832665
157	OTUD1	MAP2K2	GO:0004843 GO:0030165	6.0532	0.68610777
158	OTUD1	FLT3	GO:0004843 GO:0043621	8.4064	0.7159805
159	OTUD1	BRCA1	GO:0070536 GO:2000378	5.5968	0.67872962
160	OTUD1	CDKN1A	GO:0070536 GO:0043068	2.28	0.58854043
161	OTUD1	PIK3CA	GO:0070536 GO:0016242	1.32	0.53010702
162	OTUD1	PIK3CB	GO:0070536 GO:0006914	1.32	0.53010702
163	OTUD1	MTOR	GO:0070536 GO:0030163	4.09	0.64833381
164	OTUD1	SYK	GO:0070536 GO:0045410	2.6268	0.60334613
165	OTUD1	LPAR6	GO:0070536 GO:0051482	4.28	0.65281676
166	OTUD1	CDKN1B	GO:0004843 GO:0019903	1.48	0.5424629
167	OTUD1	FASLG	GO:0070536 GO:0097527	1.99	0.57416212

168	OTUD1	IFNAR2	GO:0070536 GO:0051607	1.32	0.53010702
169	OTUD1	PIK3CG	GO:0070536 GO:0014065	2.28	0.58854043
170	OTUD1	THBS3	GO:0070536 GO:0007160	4.09	0.64833381
171	OTUD1	COMP	GO:0070536 GO:0035264	4.09	0.64833381
172	OTUD1	VEGFC	GO:0070536 GO:0002052	1.32	0.53010702
173	OTUD1	FLT3LG	GO:0004843 GO:0030971	1.48	0.5424629
174	OTUD1	TSC2	GO:0070536 GO:1901525	1.32	0.53010702
175	OTUD1	GSK3B	GO:0070536 GO:0006983	2.6268	0.60334613
176	OTUD1	JAK3	GO:0004843 GO:0019903	6.3344	0.69033918
177	OTUD1	EFNA3	GO:0070536 GO:0007267	1.32	0.53010702
178	OTUD1	EFNA4	GO:0070536 GO:0030316	1.32	0.53010702
179	OTUD1	EFNA5	GO:0070536 GO:0051965	3.35	0.62832665
180	OTUD1	COL4A4		4.09	0.64833381
181	OTUD1	ITGA8	GO:0070536 GO:0030030	4.09	0.64833381
182	OTUD1	PRKAA2	GO:0004843 GO:0035174	1.9536	0.5722007
183	OTUD1	GYS2	GO:0070536 GO:0009749	1.32	0.53010702
184	OTUD1	FGF8	GO:0070536 GO:0007369	1.32	0.53010702
185	OTUD1	LAMB2	GO:0070536 GO:0007528	2.28	0.58854043
186	OTUD1	ITGA1	GO:0004843 GO:0019903	1.9536	0.5722007
187	OTUD1	PTEN	GO:0004843 GO:0004438	4.1952	0.65084434
188	OTUD1	IL2	GO:0004843 GO:0005125	1.48	0.5424629
189	OTUD1	YWHAG	GO:0004843 GO:0030971	6.0532	0.68610777
190	OTUD1	YWHAE	GO:0004843 GO:0046982	8.4064	0.7159805
191	OTUD1	GNB1	GO:0004843 GO:0051020	1.9536	0.5722007
192	OTUD1	GNB2	GO:0004843 GO:0051020	1.9536	0.5722007
193	OTUD1	GRB2	GO:0004843 GO:0019903	1.9536	0.5722007
194	OTUD1	RAC1	GO:0004843 GO:0031996	1.84	0.56582025
195	OTUD1	PPP2R2A	GO:0004843 GO:0051721	1.9536	0.5722007
196	OTUD1	GNG5	GO:0004843 GO:0030165	1.48	0.5424629
197	OTUD1	PPP2CA	GO:0004843 GO:0046982	1.48	0.5424629
198	OTUD1	RELN	GO:0070536 GO:2000969	3.35	0.62832665
199	OTUD1	PPP2R2B	GO:0070536 GO:0006915	1.32	0.53010702
200	OTUD1	CDK6	GO:0070536 GO:0060218	3.35	0.62832665
201	OTUD1	MDM2	GO:0004843 GO:0008097	6.164	0.68780183
202	OTUD1	COL4A3	GO:0070536 GO:0006919	3.35	0.62832665
203	OTUD1	MAP2K1	GO:0004843 GO:0047485	1.9536	0.5722007
204	OTUD1	TEK	GO:0070536 GO:0001934	2.28	0.58854043
205	OTUD1	CREB5	GO:0070536 GO:0045893	2.28	0.58854043
206	OTUD1	RELA	GO:0004843 GO:0047485	1.48	0.5424629
207	OTUD1	YWHAH	GO:0004843 GO:0046982	1.48	0.5424629
208	OTUD1	PTK2	GO:0004843 GO:0019903	3.3744	0.62906239
209	OTUD1	PPP2R3A	GO:0070536 GO:0090263	1.32	0.53010702

210	OTUD1	BCL2L1	GO:0004843 GO:0046982	1.9536	0.5722007
211	OTUD1	MCL1	GO:0004843 GO:0008320	1.9536	0.5722007
212	OTUD1	SOS1	GO:0004843 GO:0046982	4.958	0.66714635
213	OTUD1	SOS2	GO:0004843 GO:0046982	4.958	0.66714635
214	OTUD1	RBL2	GO:0004843 GO:1990841	4.958	0.66714635
215	OTUD1	PRKAA1	GO:0004843 GO:0035174	1.9536	0.5722007
216	OTUD1	EIF4EBP1	GO:0004843 GO:0051721	1.48	0.5424629
217	OTUD1	ITGA7	GO:0070536 GO:0007160	1.32	0.53010702
218	OTUD1	LAMB3	GO:0070536 GO:0050873	2.28	0.58854043
219	OTUD1	LAMC2	GO:0070536 GO:0008045	1.32	0.53010702
220	OTUD1	COL4A6	GO:0070536 GO:0007155	2.28	0.58854043
221	OTUD1	COL9A2		1.32	0.53010702
222	OTUD1	PPP2R5D	GO:0004843 GO:0004721	1.48	0.5424629
223	OTUD1	PPP2R5A	GO:0004843 GO:0004721	1.48	0.5424629
224	OTUD1	ERBB4	GO:0070536 GO:2000010	3.35	0.62832665
225	OTUD1	ANGPT1	GO:0004843 GO:0030971	6.3344	0.69033918
226	OTUD1	STK11	GO:0004843 GO:0002039	3.3744	0.62906239
227	OTUD1	LAMA4	GO:0070536 GO:0045995	2.28	0.58854043
228	OTUD1	PKN1	GO:0004843 GO:0035402	1.9536	0.5722007
229	OTUD1	PKN2	GO:0004843 GO:0042826	1.9536	0.5722007
230	OTUD1	CDC37	GO:0070536 GO:0098779	1.32	0.53010702
231	OTUD1	NTRK2	GO:0004843 GO:0005030	1.9536	0.5722007
232	OTUD1	LAMA3	GO:0070536 GO:0045995	1.32	0.53010702
233	OTUD1	CRTC2	GO:0070536 GO:0051289	2.28	0.58854043
234	OTUD1	THEM4	GO:0070536 GO:1902108	1.99	0.57416212
235	OTUD1	PPP2R2D	GO:0070536 GO:0051301	1.32	0.53010702
236	OTUD1	CREB3L3	GO:0004843 GO:0035497	1.48	0.5424629
237	OTUD1	PKN3	GO:0070536 GO:0007165	1.32	0.53010702
238	OTUD1	PIK3AP1	GO:0070536 GO:0034162	1.32	0.53010702
239	OTUD1	PHLPP2	GO:0070536 GO:0051898	2.28	0.58854043
240	OTUD1	CREB3L2	GO:0004843 GO:0035497	1.48	0.5424629
241	OTUD1	MAGI2	GO:0070536 GO:0038180	6.5664	0.69366871
242	OTUD1	RPTOR	GO:0070536 GO:0030307	1.32	0.53010702
243	OTUD1	CREB3L4	GO:0004843 GO:0035497	1.48	0.5424629
244	OTUD1	PIK3R5	GO:0070536 GO:0014065	1.32	0.53010702
245	OTUD1	PIK3R3	GO:0070536 GO:0002042	4.09	0.64833381
246	OTUD1	TSC1	GO:0004843 GO:0047485	1.9536	0.5722007
247	OTUD1	LPAR1	GO:0004843 GO:0030165	3.3744	0.62906239
248	OTUD1	TNR	GO:0070536 GO:0051968	4.09	0.64833381
249	OTUD1	BAD	GO:0004843 GO:0043422	1.9536	0.5722007
250	OTUD1	PPP2R3C	GO:0070536 GO:0032147	2.28	0.58854043
251	OTUD1	CREB3L1	GO:0004843 GO:0035497	1.9536	0.5722007

252	OTUD1	SGK3	GO:0070536 GO:2001240	2.28	0.58854043
253	OTUD1	MAGI1	GO:0070536 GO:0065003	3.8016	0.64105968
254	OTUD1	CSF3R	GO:0070536 GO:0006952	1.32	0.53010702
255	OTUD1	OSMR	GO:0070536 GO:0034097	1.32	0.53010702
256	OTUD1	LPAR4	GO:0070536 GO:0051482	1.32	0.53010702
257	OTUD1	ATF6B	GO:0004843 GO:0035497	1.48	0.5424629
258	OTUD1	G6PC3		1.32	0.53010702
259	OTUD1	MLST8	GO:0070536 GO:0031929	1.32	0.53010702
260	OTUD1	PDGFD	GO:0070536 GO:0050730	1.32	0.53010702
261	OTUD1	FGF23	GO:0070536 GO:0071374	1.32	0.53010702
262	OTUD1	GNB4	GO:0070536 GO:0006457	1.32	0.53010702
263	OTUD1	LPAR2	GO:0070536 GO:0007202	1.32	0.53010702
264	OTUD1	SGK2	GO:0070536 GO:0006979	3.35	0.62832665
265	OTUD1	G6PC2	GO:0070536 GO:0042593	1.32	0.53010702
266	OTUD1	PDGFC	GO:0070536 GO:0007171	1.32	0.53010702
267	OTUD1	GNG12	GO:0004843 GO:0030165	1.48	0.5424629
268	OTUD1	RPS6KB2	GO:0070536 GO:0006412	2.28	0.58854043
269	OTUD1	LPAR3	GO:0070536 GO:0051482	1.32	0.53010702
270	OTUD1	TNN	GO:0070536 GO:0007160	1.32	0.53010702
271	OTUD1	AKT3	GO:0070536 GO:0001938	2.28	0.58854043
272	OTUD1	ANGPT4	GO:0004843 GO:0030971	1.48	0.5424629
273	OTUD1	PPP2R2C		1.32	0.53010702
274	OTUD1	IKBKG	GO:0004843 GO:1990450	8.998	0.72194821
275	OTUD1	LAMC3	GO:0070536 GO:0016477	2.28	0.58854043
